# Beyond evidence versus truthiness: toward a symmetrical approach to knowledge and ignorance in policy studies

**DOI:** 10.1007/s11077-019-09352-4

**Published:** 2019-04-24

**Authors:** Katharina T. Paul, Christian Haddad

**Affiliations:** 1 Department of Political Science, University of Vienna, Universitätsstrasse 7, 1010 Vienna, Austria; 2 Austrian Institute for International Affairs (oiip), Berggasse 7, 1090 Vienna, Austria

**Keywords:** Agnotology, Critical policy studies, Evidence-based policy, Knowledge, Ignorance, Policy sciences, Posttruth

## Abstract

Current political developments in established liberal democracies in both Europe and North America have fundamentally called into question the normative relations between truth, knowledge and politics. Whether labeled “posttruth” or truthiness, commentators lament the willful spread and deployment of nonknowledge and ignorance as important political forces. In this paper, we discuss ignorance in its strategic dimension by weaving together insights from the sociology of ignorance with a policy-scientific approach. By means of three empirical vignettes, we demonstrate that ignorance is more than the flipside of knowledge or merely its lack: it is a constitutive feature of the policy process and is thus not uniquely symptomatic of the current era. We conclude by arguing for what we call a symmetrical approach in which ignorance receives the same quality of attention that knowledge has historically received in the policy sciences. To make fully visible the different forms of ignorance that shape policy processes, policy scholars must hone their “agnoto-epistemological sensibilities” to cope with the current challenges and advance a policy science for democracy.

## The new Realpolitik of truth and its challenge to policy sciences for democracy

Values of knowledge, truth and evidence have normatively underpinned policymaking in contemporary postwar democracies: scientific expertise is valued as knowledge input for policy, and citizens expect that the best available evidence at any given moment informs the general political trajectory. Nevertheless, in the eyes of many commentators, we have entered an era of *truthiness* or posttruth (MacInytre 2018; D’Ancona 2017), where scientific experts have allegedly lost both authority (Nichols 2017) and autonomy. In this new truth regime, politics seems to have unilaterally withdrawn from the social contract and appears to operate, once again, primarily on the basis of a stubborn will to ignorance and blatant forms of denial. For instance, in 2017, the Trump Administration sent shockwaves through public health communities when it issued a list of seven keywords that the Center of Disease Control would not be allowed to use including not only terms such as “transgender” or “fetus” that are ideologically laden in the history of US politics but also the seemingly technical term “evidence based” (NY Times 2017). In other cases, political power seems to reach unabashedly into the sphere of academic freedom and the production of critical knowledge that is inconvenient for the ruling class. In Europe, Hungary’s far-right government has succeeded in pushing the liberal Central European University out of the country and produced legislation that outlaws teaching gender studies as an academic subject in universities (The Independent 2018).

What do such developments imply for our understanding of knowledge and ignorance in policy and politics? Indeed, these events not only concern democratic societies but also raise substantive concerns and challenges for the policy sciences in terms of their modus operandi and research ontologies, and the field’s role as a facilitator in promoting the design of democratic policies that serve the public interest. This paper joins others (Perl et al. 2018; Braun and Dodge 2018) in confronting these challenges. We do so by mobilizing ignorance as a central and integral category for policy analysis.

In doing so, we build on an emerging and overdue debate in this journal. As Perl et al. (2018) point out, the current climate is frequently described as one where problems of evidence—and on the flipside, problems of ignorance in various forms from bias to denial and repression—have once again begun to haunt debates on policymaking and good democratic governance. Perl et al. (2018) show that the most established policy-scientific frameworks are indeed still capable of dealing with these challenges, of discerning the difference between fact and fiction (a capacity that seems to be key in the authors’ understanding of policy sciences), and of mitigating the impact of “erroneous evidence.” The authors conclude by suggesting that “existing policy frameworks continue to remain very useful in a postfact era and require little to no change in order to generate concrete expectations about future policymaking behavior and outcomes” (2018: 595).

While we do not in principle disagree with the general argument put forward by Perl et al., we believe that the policy sciences have long underestimated, or even *ignored,* the role of ignorance in policy. A merely defensive move animated by the desire to restore the shattered fabric of science and policy is thus not enough. Instead, we suggest seizing the opportunity and taking this debate a step further by capturing the role of ignorance as a constitutive feature of policymaking rather than as something external to it. Tackling policy *ignorance* in an upfront way and placing it on par with policy *knowledge* empirically and conceptually offers an opportunity to both make sense of and confront the current Realpolitik of truth that threatens contemporary democracies.

This paper sets out to layout the groundwork for such an approach by offering insights into three empirical instances of ignorance in the policy process and by weaving together policy-scientific insights with those stemming from the emerging literature on the sociology of ignorance. Below, in section two, we briefly contextualize our concern with ignorance in the current debate on the role of evidence in policymaking. We then proceed to discuss the concept of strategic ignorance (McGoey 2012a, b) and argue for its pertinence to policy analysis. Subsequently, we offer three research vignettes from our own work to sensitize the reader to the role of willful, strategic ignorance in policymaking and to tentatively explore different mechanisms of how ignorance is mobilized and deployed strategically at different points and at different scales in policymaking and governance. Based on these insights, we then develop elements for a critical policy science with what we call “agnoto-epistemological sensibilities.” Specifically, we propose to integrate the critical epistemologies and agnotologies of policymaking into a framework that allows for jointly and symmetrically approaching questions of knowledge and ignorance in policy. We conclude by calling for an agnoto-epistemological approach in policy studies to live up to the ideal of Lasswell and to contribute to the pursuit of a rational and science-based policy sciences for democracy.

## Mobilizing evidence against posttruth: symptom or antidote?

Nonknowledge (Böschen et al. 2010) and ignorance present all-encompassing dimensions of social life and human experience but have hardly been treated systematically in policy theories. This is the case mostly because the policy sciences have been much more concerned with the role of knowledge rather than nonknowledge as input for policy, which is a bias that is shared with many other sciences (cf. Proctor and Schiebinger 2008).

The alleged crisis of expertise and knowledge and the shift toward posttruth has sparked renewed interest in the relation between knowledge and politics and between ignorance and evidence. Reactions to this shift can be grouped into two sets of discussions. First and most prominently, we witness a renewed will to reclaim truth and evidence and to defend scientifically grounded knowledge against the perils of “truthiness” and alternative facts. This language is particularly pertinent in efforts to design effective policies to tackle climate change or vaccine hesitancy and, more generally, in the debate on the need for evidence-based policymaking (Cairney 2016; see Parkhurst 2017 for a critical review). The recent warning issued by the World Health Organization labeling vaccine hesitancy among the ten most crucial global threats speaks to this effect. Herein, *evidence* is somewhat paradoxically mobilized as an antidote to the *crisis* of evidence (cf. Kunseler and Tuinstra 2017). The emerging elite advocacy of “epistocracy” by academics such as Steven Pinker (cf. McGoey 2019) takes the rationalist stance to the extreme and risks renouncing democracy to save *Truth*.

Second and particularly in interpretive approaches to policy, we see efforts to formulate constructivist concepts of knowledge (e.g., Angermüller 2018), not least because the postmodern turn has been blamed for discrediting the notion of scientific truth (e.g., by Guardian columnist d’Ancona 2017). The academic debate on the role of evidence in politics and policy has thus reinforced two poles (Strassheim 2017; Newman 2017): while rationalists seek to use the current window of opportunity to rehabilitate the link between positive science and truth, constructivists suggest there never has been an unequivocal foundation of scientific truth claims.

This polarization has left an important blind spot that is both a root cause and symptomatic of the current crisis: the very concept of evidence and knowledge practices has been limited inasmuch as it includes only the production and accumulation of knowledge (and questions regarding its validity and significance), rather than its *absence*. That is, policy scientists, much like other scientists, have been biased in favor of knowledge rather than nonknowledge and tend to turn a blind eye to what is unknown and what has been forgotten or concealed. If discussed at all, the absence of knowledge is primarily discussed in terms of a knowledge “not yet produced” or knowledge repressed. Perl et al. (2018) take an important step by carving out space for the saliency of ignorance in their evaluation of knowledge in the current “postfact” world by drawing on the perspective of agnotology, that is, the term referring to the systematic study of nonknowledge and willful ignorance (Porter 1996: 6; see also Proctor and Schiebinger 2008; McGoey 2012a, b). They acknowledge the pervasiveness of willful ignorance in and for policymaking, yet in our reading, they remain predominantly wedded to a limited notion of ignorance: they cast ignorance, both epistemologically and normatively, as the undesired *Other* of knowledge. In this way, ignorance is assigned either a negative and neutral role (what we term “residual ignorance”) or is equated with willful manipulation and malicious lies. While these specimens of ignorance and nonknowledge surely exist, such an approach fails to engage fully with ignorance in a systematic and comprehensive fashion. Such a position, we argue, does little to help us out of the current deadlock. For what is still missing in both rationalist and postpositivist approaches to policy is a serious and rigorous engagement with different forms of ignorance at the conceptual, empirical and methodological level.

### A turn to strategic ignorance: moving beyond negative and residual accounts of nonknowledge

Puzzled by the observation that we—as social scientists but also as societies more generally—know very little about ignorance compared to knowledge, the past decade has seen the rise of “agnotology,” or the systematic study of nonknowledge, in fields such as history, sociology and anthropology (Gross 2007; Proctor and Schiebinger 2008; McGoey 2009; Croissant 2014; Böschen et al. 2010; Sullivan and Tuana 2007). Given the sheer magnitude of ignorance in the human experience, the heterogeneity of its forms, and its implications for individual and social life, agnotology takes as a point of departure the observation that “a great deal of attention has been given to epistemology (the study of how we know) when “How or why we don’t know” is often just as important, usually far more scandalous, and remarkably undertheorized” (Proctor and Schiebinger 2008: vi). Encompassing the study of different forms, causes and mechanisms of nonknowledge and ignorance, ranging from suppression and secrecy, to neglect, bias and selectivity, agnotology is introduced as a counterweight to the “serious business” of epistemology (Proctor 2008: 1–2).

In sociology, McGoey (2007, 2012a, b) has taken crucial steps in the study of ignorance by mobilizing the Foucauldian concept of knowledge/power (Foucault 1976) as productive and generative as opposed to a notion of power that is merely extractive, repressive and essentially negative. Pioneering the field of a sociology of strategic ignorance, McGoey (2007, 2012a, b; Davies and McGoey 2012) has suggested to expand Foucault’s notion of “perspectival and strategic truth” and explore how a “will to ignorance” materializes as a strategic weapon in governmental practices (McGoey 2007: 217). McGoey’s (2007) paradigmatic case study is the politics of regulation of antidepressant drugs in the UK healthcare system. McGoey shows that ignorance of detrimental adverse effects, on behalf of the drug regulatory authorities, is not just to be understood as simple neglect or inadvertent mishap. Extrapolating from her case study, McGoey argues that “it is possible that ignorance—wielded as a strategic weapon—provides solutions to a problem unique to democracies that place a premium on public access to governmental information” (McGoey 2007: 217). From her perspective, ignorance is thus understood as a strategic good and a value that is actively pursued and produced (see also Gross 2007; Best 2012).

This conception of ignorance differs starkly from what we might term “residual ignorance,” i.e., the common and often scientist perspective on ignorance as some sort of “other” to knowledge: its blind spot or antithesis, a remnant of incomplete or biased knowledge practices. As a semiotic material entity in its own right then, ignorance should be considered a part, conceptually and methodologically, of what we discern with Foucault as the “regimes of truth” that underpin social, political and policy processes broadly understood. It is precisely this stress on the generativity and productivity of ignorance, a power calculus that may be—to paraphrase Foucault one more time—very well intentional, but perhaps nonsubjective, which makes the concept of strategic ignorance indispensable to the project of critical policy studies.

### Agnoto-epistemological sensibilities in policy studies

Our own approach to ignorance specifically builds on and further develops McGoey’s (2012a) notion of strategic ignorance by trying to develop a decidedly *symmetrical* approach to the study knowledge and ignorance. The concept of symmetry was key in the development of the “strong program” in the sociology of scientific knowledge. Discontent with the way historians of science approached truth and falsity, Bloor (1991) and others (Callon 1986) put forward the concept of symmetry. Symmetry as a methodological principle suggested accounting for truth and falsity of scientific theories in the same conceptual terms. The background of this intervention was the observation that historians of science had not felt the need to develop sociological explanations for theories that were held *true*. In contrast, the allure of theories that were later proven *false* has been explained through sociological categories: reputation, interest, power, and the like. With the further development of the discipline of science and technology studies (STS), the principle of symmetry was integrated as one of the three key pillars into the “sociology of translation” (also known as Actor-Network-Theory) that was developed by Michel Callon and others (Callon 1986; Law 2004). For Callon, general symmetry explains conflicting viewpoints of different parties involved in sociotechnical disputes in the same conceptual terms, which also means developing an analytical language that comes to conceptual terms with both camps, i.e., by offering symmetrical accounts.

This notion of symmetry, we believe, offers a useful way to discuss ignorance and knowledge in policymaking. A symmetrical approach means doing away with the concept of nonknowledge as inactive and neglected “residual ignorance” and refraining from scandalizing ignorance *ex ante* in terms of malice or manipulation. Like knowledge, ignorance is then analyzed as an active and serious product that results from a variety of sociotechnical practices. Like knowledge, ignorance takes on different forms and means different things to different people and in different contexts. Like knowledge, ignorance emerges in particular infrastructures and is at once maintained by them. Like knowledge, ignorance is analyzed as a material good that can become commodified, privatized, circulated and diffused, and “weaponized” as a strategic arm in policy disputes by governmental and nongovernmental actors alike (such as members of the industry but also NGOs, see Benjamin 2018). Moreover, on a more philosophical level, symmetry prompts us to complement our heavy focus on epistemology (the systematic study of how we know) with agnotology (the systematic study of how we do not know) and hence to develop “agnoto-epistemological sensibilities” within the policy sciences.

## Strategic ignorance in policymaking: three sensitizing fieldwork vignettes

Where and how do forms and practices of strategic ignorance come to matter in policymaking? To better illustrate how a notion of strategic ignorance can help policy scholars understand ignorance in and for policymaking, we present three case vignettes from our own research. Our vignettes are rooted in past and ongoing research in the domains of health and medicine policy and concern: (1) the maintenance of convenient uncertainty in assessing vaccination programs; (2) the convenient skimming of research complexity in environmental and health standards for product label regulation; and (3) the systemic asymmetries in clinical knowledge on medication and drug prescription.^1^

### Vignette 1: convenient uncertainty in evaluating vaccination policy

National immunization programs have been under particular scrutiny in commentaries on “posttruth politics,” with vaccine skepticism depicted as one of its prime symptoms. While in this public discourse the lack of scientific literacy is problematized as a malignant form of public ignorance, other forms of ignorance have gone unnoticed, such as how evidence becomes filtered and used to inform policy. Data infrastructures known as vaccination registries appear to be key instruments that guide these decisions as they are used for epidemiological surveillance, monitoring, enabling early interventions in the case of an epidemic, and policy evaluation. Registries are thus not only technical but also sociopolitical tools. In an ongoing research project, we discovered early on that individual-level data on vaccination was either not collected or not shared across regions in Austria in the current case study. Therefore, there is a remarkable absence of data (and knowledge) on the ways in which residents make use (or not) of public vaccination programs, and this makes any comprehensive evaluations of vaccination policy fairly difficult. This absence of data seems in stark contrast to the prominence of vaccine uptake rates in policy discourse and media reports. As statisticians and epidemiologists have reported in the study, the data collected in Austria, even when aggregated, are imprecise at best. While a number of disparate actors, such as women’s groups concerned with the vaccine against human papillomavirus (HPV), epidemiologists, and medical doctors, have urged for political action to harmonize these divergent registries, government and local officials privilege financial considerations in their accounts: a central digital registry that allows for individual-level data would be too expensive.

Notwithstanding the evident financial intricacies of public health policy in federalist settings, we propose that a central data infrastructure would not only generate financial but also symbolic and political costs but would also facilitate a more comprehensive and detailed evaluation of not only individual new vaccines but policy instruments. Harmonization would lead to more effective monitoring of vaccine behavior across regions but also to more effective performance of the national immunization program overall, including better data on the compliance of medical professionals with vaccination guidelines. Indeed, we see such a model used in contexts such as Denmark, Finland, and the Netherlands, and Austria has a generous public health system that is similar to those of its European counterparts. To be clear, we cannot discern an intention to deliberately ignore precision as a rationale of public health policymakers in Austria. Nevertheless, the inertia encountered in this case illustrates a type of strategic ignorance that we might describe as a “convenient uncertainty.” The fractures in the vaccination data landscape are neither the outcome of a strategic agenda nor merely accidental. Rather, it appears as an institutionalized and ritualized form of neglect that seems to be serving a different political calculus. It is thus here that we see a need for the kind of policy research that we propose: to focus not only on what and how policymakers know but also on the kind of knowledge that is *absent* and how it is possible that they (oftentimes quite easily) do *not* know. This form of ignorance is different from those discussed in Perl et al. (2018): it does not originate from malicious intent but from agnotological convenience.

### Vignette 2: convenient complexity skimming in standards making: from knowledgeable research dossier to an ignorant “one-pager”

In earlier research, we were interested in regulatory policymaking, particularly with regard to how environmental and health safety standards are negotiated and settled in EU governance processes. For this reason, we met with Natasha, a lobbyist at a Brussels-based environmental NGO, who offered an intriguing account of how strategic ignorance is produced and processed in the deliberations on regulatory standards.

To determine the threshold values for health and environmentally friendly product characteristics, industry lobbyists commission scientific studies that assess the “life cycle” of a particular product at stake (shampoos, detergent, dishwashers, etc.). The results of these studies are a lengthy research report that typically, when conducted rigorously and responsibly, includes several paragraphs concerning the limitations of the study (as any good research report typically would). As this research report travels up the politico-administrative ladder, it becomes infused with different meanings and, more simply, it becomes shorter. Therefore, the originally extensive, even if *always*-*already* selective, body of evidence is handed over to decision makers in what Natasha refers to as a “one pager,” which features an agreeable graph of the product life cycle (with vivid colorful bars, pie charts and trend lines). What is lost in the process is not only the complexity of the study but also information on its biases and selectivity, some of which *was* originally discussed in the aforementioned sections on methodological limitations and/or guiding research assumptions.

As Natasha’s account makes clear, the industry does not actively interfere with the results, and the study does not necessarily lack methodological quality. Rather, strategic ignorance is produced in the course of information processing and an apparently “necessary” abstraction in the service of complexity reduction to provide a handy evidence base for decision makers. The crucial point, however, if we are to believe our NGO-based informant, is that industry lobbyists are well aware of this apparently naturally occurring process of skimming the complex report and hence happily “play along” in the language game of science-based policymaking. Paradoxically, their *knowledge* of how convenient ignorance is produced along the way in the policy process—somehow “nonsubjectively,” i.e., without malicious intent and willful action—and the structural frameworks that govern these kinds of governance processes constitute powerful strategic resources in the “negotiations” over desirable evidence-based standards. Moreover, this strategic move is particularly effective because it is exacerbated in this particular example by material resource differentials: as Natasha recounts, these processes typically involve 2–3 bureaucrats, 6–10 industry-based lobbyists, and Natasha as the *only* NGO-based lobbyist. On top of this, industry lobbyists hold substantial budgets at their disposal to commission various different scientific studies (that eventually turn into several handy “one-pagers”), whereas NGOs only have sufficient resources to finance one or two at most.

### Vignette 3: systemic ignorance as an effect of epistemic particular interests: pharmaceutical nonknowledge beyond scientific bias and willful manipulation

So far, we have illustrated how different forms of ignorance come to matter in public policy infrastructures and how convenient ignorance emerges in the policy process due to factors of organizational complexity reduction. The following case illustrates how strategic ignorance takes shape at the systemic macrolevel of society as an aggregate effect of various strategic micro- and mesolevel decisions regarding knowledge production. Let us consider, once again, the example of pharmaceutical regulation and the concerns over drug safety and efficacy.

Clinical trials are systematic studies typically run by pharmaceutical companies that produce data on a drug’s safety and efficacy to establish an evidence base serving clinical and regulatory decision making. However, as critical scholarship has shown, the contemporary political economy of clinical trials offers multiple ways for inscribing bias, selectivity and ignorance into the trial design (Will and Moreira 2010; Abraham 2008; McGoey 2012a, b). In this literature, the analysis often focuses on a particular clinical trial and/or the regulatory science interface between drug developers and authorities. While this scholarship at the micro- and mesolevel has been essential to rethinking the role of Big Pharma, we want to point to yet another type of ignorance produced at a different, *systemic* level.

Reading Dumit’s (2012) seminal ethnography of pharmaceutical value creation through the lens of strategic ignorance (McGoey 2012a, b) helps to demonstrate how ignorance is produced in the translation process from the individual to the systemic level of knowledge production and circulation. Due to their high costs, most clinical trials are run by private corporations and only an infinitesimal share of clinical trials is mainly sponsored by *public* health agencies. In pharmaceutical R&D departments, tough choices are made to determine where to allocate money, that is to say, which clinical trial is going to be funded and which is *not*. As pharmaceutical companies are first and foremost capitalist enterprises that are compelled by the imperatives of profit, growth, and shareholder value, research decisions are closely tied to economic calculations of the return on investment; put differently, the priority levels of competing epistemic interests are adjudicated by the firms’ assessments of financial profitability. Consequently and unsurprisingly, clinical trials that might expand a drug market are thus preferred over those that might narrow its market. In other words, this means that many studies are funded to investigate whether and when exactly it is advisable to commence or continue medication in a given direction, but only a few studies explore the rationale for discontinuing medication (i.e., the clinical rationale to *stop* treatment). For sure, there *are* clinical trials that investigate the latter, and recent trends toward valuing the effectiveness of treatments paid for by public health systems further direct research interests in this direction. However, given the sheer number of corporate-run trials and their role in the expansion of drug markets, medical professionals and society as a whole come to know much more and have much better information about when to begin or continue a medication and much less about when to discontinue medication even when the knowledge of both is equally relevant from a scientific, clinical and public point of view.

This selectivity in research interests at the micro-level of the individual company translates into a broadscale ignorance at the systemic or macrolevel of medical and public health knowledge and has vast implications for public health policy.

### Extrapolating conceptual sensibilities for strategic ignorance

In our own work, these vignettes have constituted “ethnographic moments,” or research epiphanies, that have brought the problematics of knowledge and ignorance to our attention. As policy scholars, we have mostly paid attention to questions of *knowledge* practices in policymaking and were thus puzzled not only by the ways in which *nonknowledge* and *ignorance* are generated and channeled into strategic deployments to serve particular interests and by how closely policy ignorance is related to the production of policy knowledge. Across the cases, two conceptual points that correspond to the concerns raised by sociologists of ignorance are paramount for laying the groundwork for advancing an analytical approach to policy ignorance.

First, the forms of ignorance explored here have almost nothing to do with any kinds of misinformation, manipulation, malice, “truthiness,” or alternative fact-making; that is to say, those phenomena that seem to haunt contemporary policymaking and policy sciences, respectively. Second, whereas ignorance has commonly been conceptualized mainly in terms of a negation as the undesired “void” of knowledge, these cases show that ignorance is far from a mere “residue” of epistemic neglect or of “knowledge not yet produced.” In contrast, the vignettes point to the very tangible effects of selective processing of research-based knowledge, in which ignorance emerges as a by-product in knowledge-making processes. It seems fruitful therefore to conceptualize ignorance as *a product* and a valuable good that has a materiality on its own.

As such, ignorance precisely amounts to, following and going beyond Foucault, a *form of power* complementary to the notion of productive and dispersed “knowledge/power” that is at the same time “intentional, but nonsubjective” (Foucault 1976). We hold that this nonsubjective dimension of strategic ignorance is key to understanding both its workings and its productivity as a strategic resource, or as McGoey describes it, a “strategic weapon” in the complex battles that shape both the form and the material substance of public policies; or to put it in Lasswell’s famous words, the battles that determine “who gets what when and how” that characterize the politics of policymaking.

Having laid out these ethnographic vignettes and their conceptual value, we next contrast them with how policy scholars have dealt with questions of knowledge and ignorance and highlight the blind spots that the epistemological bias of the discipline has produced. In doing so, we seek to provide the basis for a long-needed discussion within the field and to show the value of agnoto-epistemological sensibilities for policy scholarship.

## Knowledge and ignorance in policy studies: conceptual lineages and their limits

### Knowledge: policy studies and the serious business of epistemology

What scholars of agnotology have identified as involvement in the “serious business” of epistemology (Proctor 2008: 1) has also shaped the policy sciences in crucial ways. For one of the founding scholars of the policy sciences, Lasswell (1971), the discipline of policy science, is to produce and apply *knowledge of* and *in* policy, thereby enhancing the effectiveness of science-based knowledge in the policy process. While Lasswell’s approach was fundamentally designed to be in the service of democracy and active citizenship, it was later *scienticized* by what Hoppe (1999) calls neopositivist policy analysis that sought to apply scientific causal laws and formal modeling to policy analysis. In this second generation of scholarship, the policy process was conceptualized in a linear if not mechanistic fashion and was characterized by leading from problem identification to the definition of solutions, formation of adequate knowledge, and implementation of particular solutions (Lindblom 1993). This *neopositivist* generation of policy sciences came to dominate the discipline and its institutional venues, thus leaving hardly any little space for more interpretive understandings of policy embedded in broader sociocultural systems of meaning and interaction. Instead, positivist research methodologies that were backed up by theories of rational agents became mainstream in policy analyses despite their limited capacity to address complex social interactions in a meaningful way.

Nevertheless, shifts associated with various “turns” (linguistic, cultural, practice, etc.) in the social sciences and humanities triggered a gradual problematization of the notion of knowledge. This momentum was also taken up by policy analysts (Gottweis 1998) who sought to move beyond metaphysical and positivist approaches (Gottweis 1998; Fischer and Forrester 1993; Yanow 1996; Healy 1997). For policy studies, this was not merely an intellectual project in the context of the postmodern condition (Lyotard 1979) but had a practical justification: incorporating meaning-making through a sensitivity to discourses, narratives and political metaphors would allow, almost paradoxically, for more *realistic* or comprehensive accounts of policy than those emerging from rigid cost–benefit models of the neopositivist policy analysts (Gottweis 1998; Fischer 2003). These intellectual developments animated a wider rethinking of the conceptual relationships between policy and knowledge over the past three decades, including a discussion of the very ontological and epistemological foundations of policy analysis. The ambition was not, as Torgerson puts it, “to abandon inquiry, or expertise, but to appreciate limits and to suggest, as a remedy, an emphasis on deliberation, participation, and the significance of disputes among experts” (Torgerson 2017). As a result, the policy process was reconceptualized in a much more dynamic fashion with greater attention paid to the role of language, discourse, and problematizations (cf. Bacchi and Goodwin 2016). What is now known as critical policy studies emphasizes the radical contingency of both policies and the knowledge we produce in studying them (Fischer et al. 2017; Voß and Freeman 2016; Wagenaar 2011).

Building on poststructuralist theories and particularly on the work of Michel Foucault, knowledge is then no longer conceived of as the *Other* of power, and its antidote, a notion best encapsulated by Wildavsky’s (1979) notion of “speaking truth to power.” Rather, knowledge is reconceptualized as one particular form of power: knowledge is a discursive and performative force that is selectively available and appropriable in complex battles. This rearticulated notion of power has proved particularly relevant for an understanding of the political role of scientific expertise in the policy process and has helped to make mainstream an understanding of scientific expertise as socially and institutionally embedded (Hoppe 2005; Jasanoff 1990, 2011; Nutley et al. 2010; Strassheim and Kettunnen 2014). This has led to a new qualification and broadening of the concept of “evidence” in and for policy, and includes practices other than scientific output, such as experiential knowledge (cf. Fleming and Rhodes 2018) and local knowledge (Yanow 1999). To summarize, policy scientists have developed critical understandings of the notion of knowledge and its manifestations in power relations but have simultaneously held on to the notion of ignorance (or nonknowledge) as its inferior *Other*.

### Ignorance: accounting for (residual) ignorance in policy analysis

To be sure, ignorance has been a concern for policy analysts yet in a very different sense than that suggested by the emerging literature on agnotology and strategic ignorance. For those familiar with rational choice models, it comes as no surprise that ignorance has been a major theoretical concern in the positivist and rationalistic studies of political behavior and decision theory. In these theories, however, ignorance has been mostly conceptualized through microeconomic lenses in terms of an actor’s lack of knowledge in the context of a benefit–cost analysis (i.e., what we have termed “residual ignorance”). Down’s (1957) “Economic theory of democracy” is a foundational text in the formalist appreciation of ignorance, understood in terms of informational barriers and asymmetries. Clearly, the use of ignorance for the rational actor is also strategic but only to the extent that he/she actively (and somewhat deliberately) chooses which type of information (e.g., substantive knowledge of party ideologies or the ramifications of a given policy) is sufficiently utile given the pressure of scarce resources of time and attention. This model presumes that the selection of information produces ignorance, which is based on an implicit cost–benefit analysis of the individual actor animated by the transitive hierarchy of preferences and by the principles of decisional efficiency (Downs 1957). Following up on this model, political scientists and policy analysts have problematized ignorance as a lack of information somewhere between the two poles of a deficit model in which ill-informed citizens signify a failure of democracy on the one hand and conceptions of “rational ignorance” on the other (see, e.g., Gilens 2001).

The conceptual understanding of ignorance we seek to introduce in the policy sciences is also notably different from the various concepts developed in the critical policy studies tradition, which encompasses a range of positions, from critical realism to poststructuralism (cf. Fischer et al. 2017). In poststructural theories, ignorance is mostly couched into the notions of uncertainty and contingency. In particular, the poststructuralist school is concerned with ignorance as an irreducible contingency and a blind spot in human experience (Howarth et al. 2000; Glynos and Howarth 2007). In this regard, notions such as the Derridean *diffèrance* or the Lacanian “Real” are understood as those dimensions of experience that resist and subvert any positive determination through signification and symbolization, thus constituting that which is *always*-*already* external to positive knowledge and discourse. Nevertheless, while these concepts provide invaluable insights into the limits of knowledge, they are of little help for conceptually grasping and analyzing practices of active and willful ignorance in policymaking. Likewise, critical realist and historical materialist policy analyses try to approach nonknowledge through concepts of hegemony and strategic selectivity (e.g., Vadrot 2016; Brand and Vadrot 2013).

This brief review indicates that all of the prevailing approaches remain wedded to the notion of “residual ignorance” delineated above and cannot fully capture the full generativity and productivity of strategic ignorance. All these variants of ignorance in policy studies are grounded in a framework of political epistemology and treat ignorance merely as the flipside of knowledge. The focus is predominantly on *knowledge*, and where ignorance is discussed, it is understood as the result of epistemic neglect, a lack of will to gain knowledge, an irreducible blind spot of human subjectivity, or as a repressed form of knowledge. As such, these theories share an (at least implicit) normative assumption, deeply rooted in the logocentric tradition of European philosophy, that knowledge—and knowledge alone—can serve as the norm and the foundation of public policy.

In contrast, we argue for a shift toward a perspective that decenters knowledge and epistemology in favor of a more *symmetrical* approach to both (different forms of) knowledge and (different forms of) nonknowledge, which is captured by the (admittedly clumsy) notion of *agnoto*-*epistemological sensibility*. In our view, practices of ignorance-making are intrinsic to the policy process as much as any knowledge-making practices. Policy analysts must find ways to make the production of ignorance analytically visible as an essential and sometimes even constitutive practice of policymaking irrespective of the different forms that ignorance takes in a particular case (political convenience, knowledge filtering, or systemic design).

## Lasswell’s legacy: how a symmetrical approach can serve democracy

This paper takes as a point of departure the proclaimed rise of “truthiness” in politics and policy for an emerging and overdue discussion of the role of nonknowledge and ignorance in the policy process. While most established policy-scientific frameworks can account for misinformation and truthiness as seemingly exogenous variables interfering in democratic politics (Perl et al. 2018), we argue that policy analysis remains fundamentally grounded in a knowledge-centric political epistemology and is thus unable to account for the active production of ignorance as an essential and constitutive feature of policymaking, and the politics and power struggles shaping it. To shed light on the latter, we showcased three research vignettes that point to the role of ignorance as part of what Hall (1993) would call “normal politics” and used these to advocate a policy-scientific inquiry that puts ignorance and nonknowledge on par with knowledge. The three kinds of ignorance we identify are notably different in quality and cannot be accounted for by presuming malicious intent, concealment, or repression of knowledge. Instead, we show that ignorance appears in much more subtle and systemic ways: data may not be collected or evaluated out of political convenience of preserving the status quo; knowledge may be filtered when scientific evidence travels up the politico-administrative ladder in environmental assessments; or ignorance and nonknowledge may be embedded in institutional systemic design that is biased in favor of vested interests. Our vignettes are not meant to be exhaustive illustrations but rather paradigmatic examples from policy-analytical research.

The relevance of our discussion lies not in its novelty, as the basic groundwork has been laid in the neighboring disciplines of sociology (McGoey 2007, 2012a, b; Gross 2007; Kelly and McGoey 2018) and history of science (Proctor and Schiebinger 2008). Instead, its pertinence is in its urgency and the current opportunity to intervene as policy scholars: understanding the role of strategic, convenient, and systemic ignorance in the policy process is key to tackling the current Realpolitik of posttruth. If today’s predicament of posttruth is to be taken seriously analytically and politically, then symmetrical policy studies allow us to discern the regimes of truth by analyzing the particular forms and practices of both knowledge and ignorance that are selectively naturalized, incentivized, problematized, etc. The current sense of crisis (of science and politics) has produced a kind of “institutional void” (Hajer 2003) in which scientific experts no longer hold unquestioned authority, but in which policy scholarship may be well equipped to take on the kind of role that Lasswell had foreseen for it.

Doing so, however, requires recalibrating our research frameworks in order to arrive at a more realistic, more useful, and more symmetrical (Bloor 1991; Callon 1986; Law 2004) policy science. To this end, we need to rethink the very epistemologies of science or evidence-based policy in terms of the multiple and heterogeneous knowledge practices that inform what comes to be understood as evidence. This means realizing that the challenge is not so much that “evidence becomes politicized,” as Perl et al. (2018) seem to suggest but that evidence-based policy needs to move beyond its current technocratic shape and integrate different forms of knowledge to provide a more comprehensive and symmetrical account of the politics of knowledge production (see also Wieringa et al. 2017). Such an overhaul of the normative epistemic underpinnings of contemporary policymaking is beyond the scope of this paper, but both as a political toolbox and rhetoric in the current political climate, evidence-based policy will only stand a chance if the very notion of evidence is framed first to more symmetrically include ignorance and second to more comprehensively include different forms of knowledge.

We hope that the present discussion may serve as one of many elements in this endeavor. What we call agnoto-epistemological sensibilities can complement any existing models of policy analysis (be it multiple streams, policy cycle, or advocacy coalitions, or those working with interpretive approaches). Ignorance takes on manifold shapes, is articulated differently across arenas, and cannot be captured in a single unifying framework or method. Instead, discerning and making sense of the powers of ignorance in policymaking and governance may require different methodologies depending on the context in which the research takes place. In some cases, this may require more ethnographic comparative work (cf. Simmons and Smith 2017), and in other cases, this may require data reanalysis.

Since posttruth has not cropped up instantly and unexpectedly with the election of current populist politicians or with the emergence of rightwing populist movements and parties but has a long and protracted genealogy, we must prepare for a long and protracted battle to reclaim scientific reason and evidence in policymaking in the name of democratic politics. Again, the proclaimed crisis is not new, nor does our way of thinking about truth have to be: in the interwar period, leading intellectuals such as Max Horkheimer and Antonio Gramsci considered the universality of truth in the face of rapidly proliferating fascist propaganda and mobilization (and the rampant and malicious lies that were spread in these contexts). The genuine value of their reasoning is in their dialectical approach to truth in the sense that they did not succumb to false dichotomies. They held that truth is both universal and historical, and precisely because of this concept it is vital in times of sociopolitical crisis and regressive tendencies to avouch and fight for truth. Perhaps we are once again challenged with a similar task: to reclaim scientific knowledge and evidence as steadfast democratic values for policymaking and at the same time to defend the contingency of truth regimes that are perpetuated by plural of forms of knowledge and ignorance in and for plural democracies.

## Footnotes

1 Vignette 1 is based on research conducted by the first author (KP); vignettes 2 and 3 emerged in the second author’s work (CH).
